# Allele-Skewed HLA-DR Immunopeptidomes of *Bordetella pertussis*

**DOI:** 10.3390/vaccines14090733

**Published:** 2026-08-25

**Authors:** Hooman Yari, Saghar Kaabinejadian, Ricardo da Silva Antunes, Sandra K. Armstrong, Timothy J. Brickman, Alessandro Sette, William H. Hildebrand

**Affiliations:** 1 Department of Microbiology and Immunology, College of Medicine, The University of Oklahoma Health Campus, Oklahoma City, OK 73104, USA; hooman-yari@ou.edu (H.Y.); saghar-kaabinejadian@ou.edu (S.K.); 2 Center for Vaccine Innovation, La Jolla Institute for Immunology, La Jolla, CA 92037, USA; rantunes@lji.org (R.d.S.A.); alex@lji.org (A.S.); 3 Department of Microbiology and Immunology, University of Minnesota Medical School, Minneapolis, MN 55455, USA; armst018@umn.edu (S.K.A.); brick011@umn.edu (T.J.B.); 4 Department of Medicine, Division of Infectious Diseases and Global Public Health, University of California San Diego, La Jolla, CA 92093, USA

**Keywords:** *Bordetella pertussis*, immunopeptidomics, HLA-DR, acellular pertussis vaccine, antigen presentation, peptide ligand

## Abstract

Background/Objectives: Infection with *Bordetella pertussis* causes whooping cough. CD4^+^ T cell responses depend on bacterial peptides displayed by HLA class II, yet allele- and strain-resolved maps of naturally processed *B. pertussis* HLA-DR ligands remain limited. We sought to define which antigens yield HLA-DR ligands in a human macrophage model and whether presentation is skewed by HLA-DR molecule and bacterial strain. Methods: THP-1–derived macrophages were pulsed with whole-cell lysates of *B. pertussis* vaccine/reference strain Tohama I or clinical isolate D420. HLA-DR was immunoaffinity purified; eluted peptides were identified by LC-MS/MS and assigned to HLA-DR molecules encoded by HLA-DRB1*01:01, HLA-DRB1*15:01, and HLA-DRB5*01:01. Results: We identified 63 *B. pertussis* peptide ligands from 29 source proteins. Presentation was skewed by HLA-DR molecule: DRB1*01:01 accounted for 37 ligands from 21 antigens, DRB1*15:01 for 22 from 7, and DRB5*01:01 for 4 from 4. Most source proteins contributed ligands primarily to one HLA-DR molecule, so an antigen that supplies peptides to one DR product need not supply peptides to another. Strain further partitioned the ligandome: 32 ligands unique to Tohama I, 12 to D420, and only 19 from 8 proteins with both lysates. Only three antigens contributed ligands to both DRB1*01:01 and DRB1*15:01; two of these, pertactin and filamentous hemagglutinin, are components of current acellular pertussis vaccines. Conclusions: *B. pertussis* HLA-DR ligandomes are jointly shaped by bacterial strain and HLA-DR molecule. Antigens can interact selectively with individual HLA-DR products, and peptides from a given antigen may be recovered after pulse with one strain but not another. These findings support HLA-DR and strain-aware interpretation of class II presentation and nominate BrkA autotransporter (Bordetella resistance to killing A), outer membrane protein A (OmpA), tracheal colonization factor (TcfA), and a divalent metal transporter (DMT) family transporter for follow-up.

## 1. Introduction

Whooping cough is a highly contagious, potentially life-threatening respiratory illness, especially in infants, caused by the Gram-negative bacterium *Bordetella pertussis*. Vaccination has markedly reduced disease burden, and both innate and adaptive immunity contribute after natural infection or immunization [1]. CD4^+^ T cells—particularly those with T helper 1 (Th1)– and T helper 17 (Th17)–associated programs—are thought to support durable, cell-mediated help and to orchestrate effector responses that control bacterial infection; vaccines or infection histories that elicit robust CD4^+^ immunity are therefore of central interest for long-term protection against *B. pertussis*.

Introduction of inactivated whole-cell pertussis (wP) vaccines in the 1940s dramatically reduced incidence—for example, from approximately 157 cases per million to <1 per million in the United States at peak control [2]. Because reactogenicity and manufacturing considerations favored more defined products, many countries replaced wP with acellular pertussis (aP) vaccines in the 1990s [3,4,5]. Unlike wP, which exposes the immune system to a broad mixture of bacterial material, licensed aP formulations are subunit vaccines built from a small set of purified or genetically or chemically detoxified antigens—classically pertussis toxin (PT), filamentous hemagglutinin (FHA), pertactin (PRN), and fimbrial types FIM2 and/or FIM3 (FIM), depending on the product [4,6]. The transition to acellular vaccines therefore required selecting antigens that are immunogenic and can be presented broadly across human class II HLA molecules.

Despite high pediatric coverage with aP in many industrialized countries, *B. pertussis* continues to circulate globally, with periodic increases in reported cases; infants remain at highest risk for severe outcomes [4,7,8,9,10]. Contributing factors remain debated and are likely multifactorial, including evolutionary change in circulating strains—for example, antigenic drift among surface proteins, variation or loss of vaccine-included antigens such as PRN, and divergence in toxin or adhesin sequences [11,12,13,14]—together with waning vaccine-elicited immunity that permits ongoing transmission [3,5,15]. These patterns underscore a tension between immunity focused on a narrow antigen set and a pathogen capable of antigenic change, motivating strategies to broaden or refocus the *B. pertussis* antigen repertoire addressed by vaccination.

Broadening the immune response by expanding the antigen content of acellular vaccines is a promising direction for improving efficacy [16]. Because the limited number of purified antigens in licensed aP products may be suboptimal for long-lasting protection against a continuously evolving bacterium, next-generation pertussis vaccines will likely require expanded or revised antigen sets. Candidate targets have been proposed through complementary approaches, including genome-wide or proteome-informed identification of CD4^+^ T cellreactive regions in human donors [15,17,18], *B. pertussis* proteomics [19,20,21], and serological profiling after infection or vaccination [19,22,23]. Such work generates valuable hypotheses about immunogenic proteins and candidate peptide–HLA pairs. However, for class II-restricted CD4^+^ responses, a fundamental question remains only partially addressed: which *B. pertussis* proteins give rise to peptides that are actually loaded onto specific HLA-DR molecules on human antigen-presenting cells (APCs) under defined antigen-processing conditions? Direct answers require experimental access to naturally processed HLA-DR–associated peptides—information that cannot be reliably inferred from binding predictions or from T cell assays performed in unrelated HLA haplotypes.

CD4^+^ T cell recognition of protein antigens is HLA class II–restricted: APCs degrade internalized proteins into peptides that load onto HLA-DR, -DQ, and/or -DP heterodimers for display to T cell receptors. Among these loci, HLA-DR is often the dominant restriction element for CD4^+^ responses in human populations, and allelic polymorphism at HLA-DRB1 (and linked DRB genes) changes which peptide sequences can be productively bound and presented. The HLA-DR–associated peptide repertoire on APCs is therefore a proximate determinant of which CD4^+^ specificities can be engaged during *B. pertussis* vaccination or infection. Direct identification of eluted HLA-DR ligands provides a complementary, HLA-centric foundation for interpreting CD4^+^ immunity—especially when analyzed allele by allele.

Here, we used immunopeptidomics to characterize HLA-DR–associated *B. pertussis* peptide ligands eluted from HLA-DR immunoprecipitated from THP-1–derived macrophages pulsed with whole bacterial lysate, allowing the class II APC pathway to encounter a complex, physiologically plausible mixture of bacterial proteins. We focused on three HLA-DR allotypes—HLA-DRB1*01:01, HLA-DRB1*15:01, and HLA-DRB5*01:01—and identified eluted ligands by liquid chromatography–tandem mass spectrometry. To probe robustness across genetic backgrounds relevant to epidemiology and vaccine production, we compared lysates from the laboratory/vaccine strain Tohama I [6,24,25] and the clinically relevant isolate D420 [26,27]. Our aims were (i) to define which *B. pertussis* source proteins contribute ligands to each HLA-DR molecule in this model, (ii) to determine the extent of overlap versus allele-private presentation among the three HLA-DR allotypes, and (iii) to evaluate whether presentation patterns are conserved between strains for proteins detected in both lysates—information that situates contemporary vaccine antigens within a broader, allele-aware map of macrophage class II ligandomes for *B. pertussis*.

## 2. Materials and Methods

### 2.1. Mammalian Cell Culture

THP-1 monocytes (ATCC TIB-202) were obtained from American Type Culture Collection (Manassas, VA, USA) and were grown and maintained in Roswell Park Memorial Institute (RPMI) 1640 medium (Gibco, Thermo Fisher Scientific, Waltham, MA, USA), supplemented with 10% FBS (Gibco, Thermo Fisher Scientific, Waltham, MA, USA) at 37 °C in 5% CO_2_ atmosphere. The HLA genotype of the cells was determined prior to their use in experiments to ensure authentication.

### 2.2. Bacterial Cell Culture and Preparation of B. Pertussis Lysates

The Tohama I and D420 strains of *B. pertussis* were grown in 1 L of iron-free Stainer-Scholte (SS) medium at 37 °C and cells were harvested at their late exponential growth phase by centrifugation at 8000× *g* for 10 min. The cell pellets were washed with cold PBS, weighed, and resuspended in 20 mL of cold sterile PBS. After a freeze–thaw cycle in a dry ice–ethanol bath, the bacterial cell suspensions were lysed by two sequential passages through a surface-sterilized French pressure cell at 4 °C under 20,000 psi pressure. The bacterial cell homogenates were centrifuged at 3000 × g for 10 min to remove any unbroken cells, and the supernatant fluids were transferred into fresh sterile containers. The lysates were streaked onto Bordet–Gengou (BG) agar plates to assess levels of viable bacteria remaining and other potential microbial contamination. The *B. pertussis* lysates were flash-frozen in a dry ice–ethanol bath and stored at −80 °C until use.

Before pulsing the cells, the lysates were heat-inactivated at 56 °C for 45 min and subsequently homogenized by sonication using a Qsonica Q125 sonicator equipped with a GL-10 probe (Qsonica, Newtown, CT, USA). Sonication was performed at 50% amplitude for 20 s, followed by a 20 s rest period on ice, for a total of six sonication–rest cycles.

### 2.3. Differentiation and Pulsing of THP-1

For each experiment, 4 × 10^8^ THP-1 monocytes were stimulated with phorbol 12-myristate acetate (PMA; EMD Millipore, Burlington, MA, USA) at 10 nM for 72 h to induce differentiation into phagocytic macrophage-like cells. After 48 h rest in fresh complete medium, they were stimulated with recombinant human interferon gamma (IFN-γ; Peprotech, Rocky Hill, NJ, USA) at 200 U/mL for another 48 h to induce expression of HLA-DR on the surface. For pulsing with the bacterial lysate, cells were incubated with the bacterial lysate at 1 mg/mL, while the concentration of IFN-γ was maintained at 200 U/mL. After a 24 h incubation period, cells were trypsinized, collected and washed thoroughly with phosphatebuffered saline (PBS) in three cycles of resuspensioncentrifugation. Cell pellets were stored at −80 °C before further processing.

### 2.4. Flow Cytometry

Undifferentiated THP-1 cells or differentiated THP-1 cells after trypsinization were blocked in a 5% solution of normal goat serum (Abcam, Waltham, MA, USA) in the cell staining buffer (Biolegend, San Diego, CA, USA). After removing the blocking agent, they were resuspended at 1 × 10^6^ cells in 100 µL of cold cell staining buffer and incubated with FITC- or PE-conjugated anti-HLA-DR clone L243 antibody (Biolegend, San Diego, CA, USA) and anti-CD14 antibody (Biolegend, San Diego, CA, USA) or corresponding conjugated isotypes (Biolegend, San Diego, CA, USA) for 30 min. Stained cells were analyzed for immunofluorescence using a Stratedigm S1200Ex (Stratedigm, San Jose, CA, USA). Cell debris and aggregates were excluded from the analysis by appropriate gating. Results were analyzed using the FlowJo version 10.10.0 software (Becton, Dickinson, Ashland, OR, USA).

### 2.5. Extraction of HLA-Bound Peptides

HLA-bound peptides were extracted from the cell pellets based on the previously published method with some modification [28]. Cell pellets were resuspended in a lysis buffer consisting of 50 mM Tris (pH 8.0), 150 mM NaCl, and 0.5% Igepal (octylphenoxy poly(ethyleneoxy)ethanol), supplemented with the cOmplete protease inhibitor cocktail (Roche, Mannheim, Germany). The resulting material underwent high-speed centrifugation at 200,000× *g* for a period of 90 min using a Beckman Coulter Optima ultracentrifuge (Beckman Coulter, Indianapolis, IN, USA). Following filtration, the clarified supernatants were applied to in-made immunoaffinity columns conjugated with L243 monoclonal antibodies to selectively isolate HLA-DRcomplexes. Columns were washed sequentially with a series of wash buffers as previously described [28] and were eluted with 0.2 N acetic acid after the last wash. The entire extraction process was performed at 4 °C. For denaturation of HLApeptide complexes and release of the bound peptides, glacial acetic acid was added to the eluate to a final concentration of 10% (*v*/*v*), followed by incubation at 76 °C for 10 min. Before fractionation of the extracted peptide mixtures by phase high-performance liquid chromatography (RP-HPLC), eluates were dried under vacuum using a CentriVap concentrator (Labconco, Kansas City, MO, USA) and solid residues were dissolved in water containing acetic acid (10% *v*/*v*).

### 2.6. Fractionation of HLA Peptides by RP-HPLC

To reduce the complexity of the peptide pool obtained from the affinity purification process, the samples underwent fractionation via reverse-phase high-performance liquid chromatography (RP-HPLC). Initially, a CentriVap vacuum concentrator (Labconco, Kansas City, MO, USA) was utilized to dehydrate the eluate. The remaining solid material was then reconstituted in a 10% aqueous acetic acid solution and processed using either a Shimadzu Nexera (Shimadzu Scientific Instruments, Pittsburgh, PA, USA) or a Paradigm MG4 system (Michrom BioResources, Auburn, CA, USA). The separation was performed on a Gemini C18 column (150 mm length, 5 μm particles, 110 Å pores; Phenomenex, Torrance, CA, USA). The mobile phase system, maintained at pH 2, consisted of two components: Solvent A comprised of 98% water and 2% acetonitrile (ACN), and Solvent B (95% ACN, 5% water), with both containing 0.1% trifluoroacetic acid (TFA). After the column was equilibrated to 2% Solvent B, the peptides were introduced over a 19 min loading phase at a flow rate of 160 µL/min, which was maintained throughout the entire gradient. Following sample loading, the gradient was programmed as follows: a linear increase from 4% to 40% Solvent B over 40 min, followed by a second linear increase to 80% Solvent B over 8 min, a 4 min hold at 80% Solvent B, and a return to 2% Solvent B over 3 min. Effluent monitoring was conducted by measuring ultraviolet (UV) absorbance at 215 nm, and a Gilson FC 203B collector (Gilson, Middleton, WI, USA) was used to collect discrete fractions at 2 min intervals.

### 2.7. Liquid Chromatography–Tandem Mass Spectrometry

Following the first chromatography, 19 peptide-containing fractions were evaporated to dryness and then reconstituted in an aqueous solution containing 10% acetic acid, 2% ACN, and iRT peptides (Biognosys, Schlieren, Switzerland) as internal standards. These fractions were individually injected into an Eksigent nanoLC 415 system utilizing a 0.5 mm trap column (350 µm ID) packed with 3 µm ChromXP C18 particles (120 Å pore size), followed by a 15 cm analytical column (75 µm ID) featuring the same stationary phase (AB Sciex, Framingham, MA, USA). The chromatographic separation was performed at pH 2.5 using a two-mobile-phase system: Solvent A (0.1% formic acid in water) and Solvent B (0.1% formic acid in 95% ACN). After a 2% Solvent B pre-equilibration step, peptides were loaded onto the trap at 5 µL/min and subsequently eluted through the analytical column at a flow rate of 300 nL/min. Elution was achieved via a dual linear gradient: increasing from 10% to 40% B over 70 min, followed by a ramp to 80% B over 7 min.

Peptides were introduced into an AB Sciex TripleTOF 5600 time-of-flight mass spectrometer using either a Nanospray III (AB Sciex, Framingham, MA, USA) or Digital PicoView (New Objective, Littleton, MA, USA) ion source at a potential of 2400 V. Acquisition followed a data-dependent acquisition (DDA) strategy, starting with a 0.25 s TOF-MS survey scan across the *m*/*z* 300–1250 range. For each cycle, up to 22 precursor ions—characterized by intensities exceeding 200 cps and charge states from +2 to +4—were targeted for collision-induced dissociation (CID). These MS/MS events occurred over a maximum of 3.3 s, with specific *m*/*z* values excluded for 30 s after three initial observations. Fragmentation was optimized using dynamic collision energy based on the mass and charge of the ions, and data were visualized using PeakView Software version 1.2.0.3 (AB Sciex, Framingham, MA, USA).

### 2.8. Data Analysis

To assign sequences to the resulting spectra, the PEAKS Studio 11.5 software (Bioinformatics Solutions, Waterloo, ON, Canada) was utilized. The identification process was executed with a precursor mass deviation limit of 30 ppm and a product ion mass error tolerance of 0.02 Da. A database comprised of SwissProt Homo sapiens (taxon identifier 9606, accessed on 1 May 2026) and UniProt *B. pertussis* strain Tohama I (taxon identifier 257313, accessed on 1 May 2026) or the strain D420 RefSeq protein data (RefSeq GCF_001013565.1, accessed on 1 May 2026) [27] and iRT peptide sequences was used as the reference protein database. Variable post-translational modifications including acetylation, deamidation, pyroglutamate formation, methionine oxidation, cysteinylation, and citrullination were analyzed in database search using the immunopeptidome workflow. The assigned peptide sequences were subjected to a 1% false discovery rate (FDR) threshold using the PEAKS decoy-fusion procedure, and after removing PTMs from peptide sequences, they were assigned to HLA-DR alleles by submitting to MHCMotifDecon-1.2 available via https://services.healthtech.dtu.dk/services/MHCMotifDecon-1.2 (accessed on 6 May 2026, Technical University of Denmark, Lyngby, Denmark) [29]. GraphPad Prism software version 9.0.2 (GraphPad Software, San Diego, CA, USA) was used to fit size-distribution data the lognormal distribution model to calculate peptide length geometric means. An unpaired *t*-test with HolmSidak correction for multiple comparisons (α = 0.05) was used to assess the differences in contribution of different DR alleles to total peptides and *B. pertussis* peptides. The peptide sequences were aligned human proteome using IEDB PepMatch tool [30].

## 3. Results

### 3.1. Establishment of a THP-1–Derived Macrophage Model

**Figure 1:**
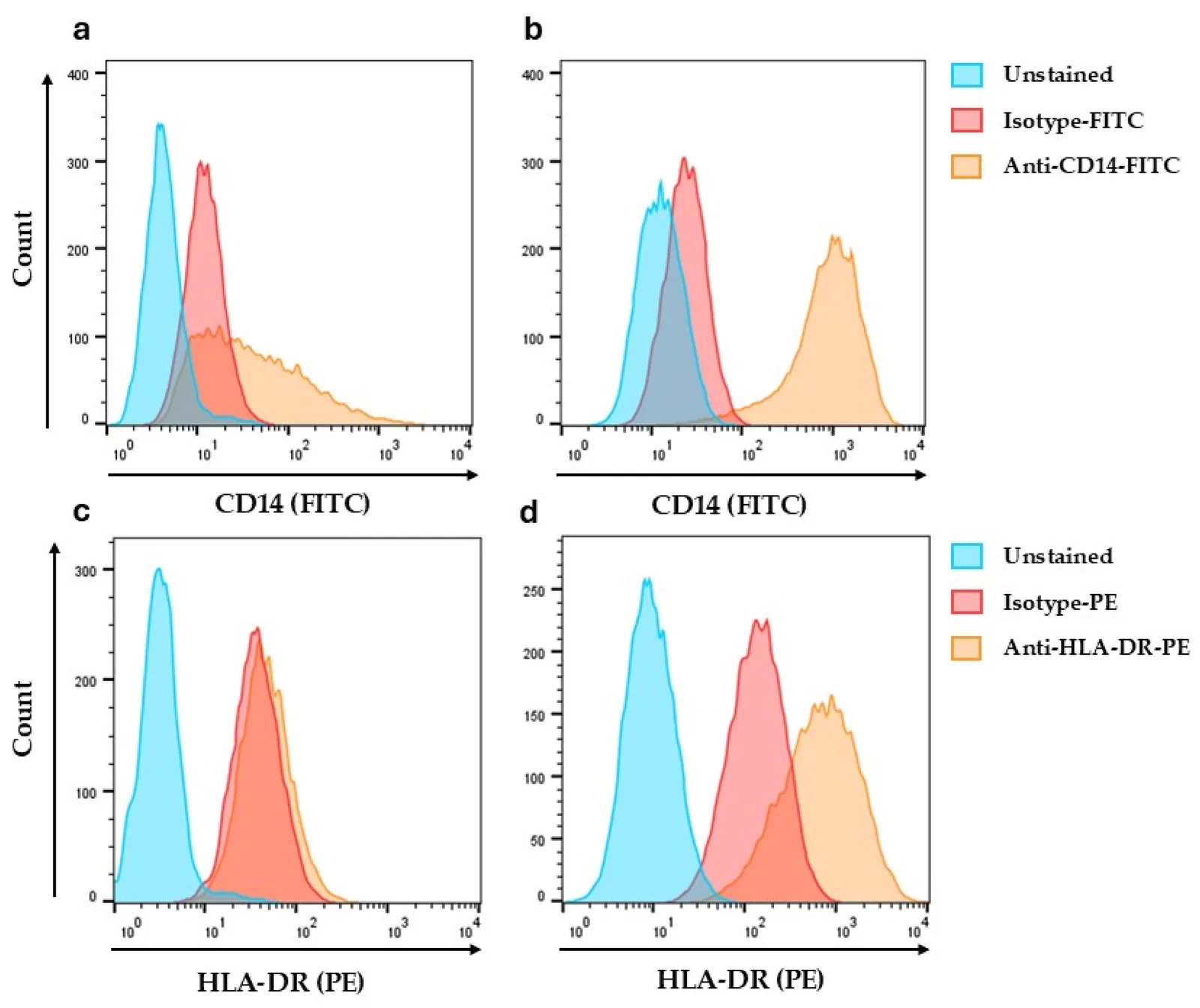
Surface expression of CD14 and HLA-DR in *naïve* monocytic THP-1 cells and *on* THP-1-derived macrophage-like cells after PMA *plus* I*FN-*γ. Overlays include the corresponding isotype-control antibody. (**a**) CD14 on monocytic cells; (**b**) CD14 on macrophage-like cells; (**c**) HLA-DR on monocytic cells; (**d**) HLA-DR on macrophage-like cells.

THP-1 differentiation into macrophage-like cells was evaluated by flow cytometry for CD14 and HLA-DR. CD14 expression increased after differentiation of monocytic cells into their macrophage-like state (Figure 1a,b). Monocytic THP-1 cells expressed a low level of HLA-DR on the surface (Figure 1c). To augment HLA-DR expression, PMA-differentiated cells were treated with IFN-γ; the macrophage-like cells shown in Figure 1b,d are this PMA plus IFN-γ condition, which markedly increased HLA-DR relative to monocytic cells (Figure 1c vs. Figure 1d). *B. pertussis* lysate-pulsing experiments were performed using cells differentiated with PMA at 10 nM concentration for 72 h duration and IFN-γ at 200 U/mL for 48 h.

### 3.2. HLA-DR-Bound Peptide Repertoire of Differentiated THP-1 Cells After B. pertussis Lysate Pulse

**Figure 2:**
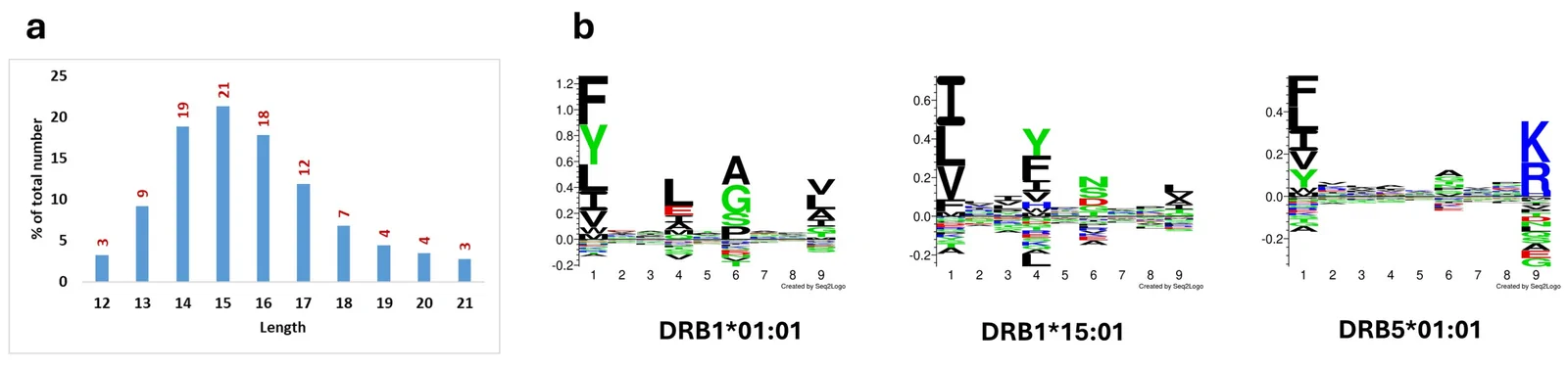
Global HLA-DR ligand characteristics after *B. pertussis* lysate pulse. (**a**) Length distribution of HLA-DR-bound peptides isolated from differentiated THP-1 cells and (**b**) binding motifs of peptides assigned to each HLA-DR molecule expressed by THP-1 cells.

After pulse with bacterial lysates prepared from Tohama I and D420, peptides were eluted from immunopurified cell-surface HLA-DR and analyzed by LC-MS/MS using the immunopeptidomics workflow described in Methods. Across both strain conditions, assignments mapped to the three HLA-DR molecules expressed by THP-1 (HLA-DRB1*01:01, HLA-DRB1*15:01, and HLA-DRB5*01:01) totaled 1587 unique sequences at a 1% false discovery rate (FDR). The length distribution peaked at 14–16 residues (Figure 2a), consistent with prior reports for HLA-DR ligands (~15 aa). Eluted peptide binding motifs (Figure 2b) matched established binding motifs for these alleles [31,32]. The *B. pertussis* assignments exhibited a minimum of three amino acid mismatches from the closest match in the human reference proteome.

### 3.3. Bordetella Pertussis–Derived HLA-DR Ligands

Using DDA and 1% FDR at the peptide level, the Tohama I strain yielded 51 *B. pertussis* peptide sequences derived from 22 *B. pertussis* proteins. Similarly, THP-1 pulsed with lysate from strain D420 provided 31 bacterial peptides derived from 17 *B. pertussis* proteins. These peptides and their corresponding source proteins are listed in Table 1.

The HLA-DRB1*01:01 presented seven GroEL-derived peptides, six peptides from the DNA-binding protein, and four peptides derived from alcaligin biosynthesis protein C (AlcC). The bacterial antigen that was most frequently sampled for presentation by class II HLA was OmpA with DRB1*15:01 presenting 16 different OmpA peptides.

**Figure 3:**
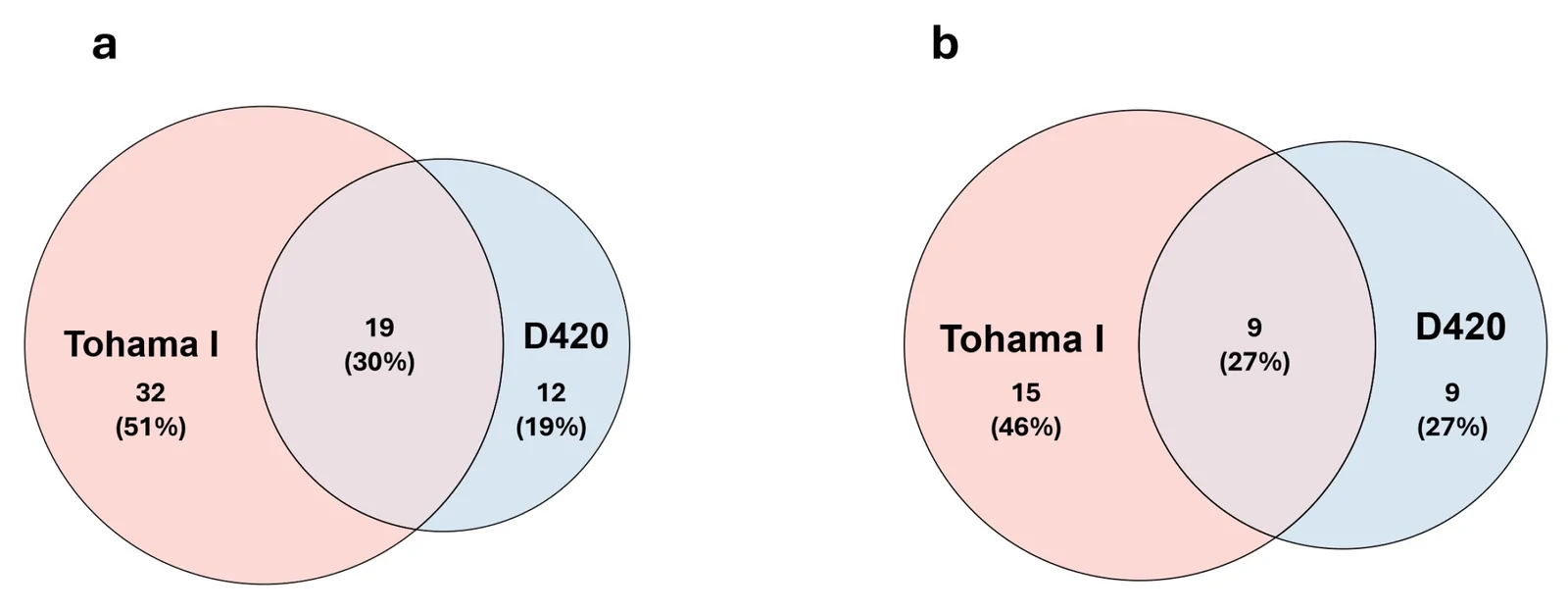
Overlap of *B. pertussis* HLA-DR-bound peptide sequences presented by differentiated THP-1 cells pulsed with Tohama I or D420 lysates. (**a**) Overlap of unique full-length bacterial peptide sequences recovered from each strain pulse. Nested length variants that share a registry are counted as separate sequences. (**b**) Overlap after collapsing nested length variants to a single 9-mer binding core per nested set, so each core is counted once.

A total of 32 unique peptides were obtained from Tohama I lysates and 12 unique peptides were found using D420 lysates. These data suggest that differentiated THP-1 cells present a greater number of peptides from Tohama I than from strain D420 (Figure 3a).

To eliminate the biases due to the length variants of a single binding core, we compared the binding core of the peptides presented from the Tohama I and D420 strains and confirmed that a greater number of peptides were presented by differentiated THP-1 cells when they were pulsed with the Tohama I compared to D420 (Figure 3b). Peptides originating from OmpA were presented by DRB1*15:01 with either Tohama I or D420, whereas DRB1*01:01 presented AlcC-derived peptides. The DNA-binding protein was presented only with Tohama I lysates, and more peptide ligands from chaperonin GroEL were presented by DRB1*01:01 after pulsing with Tohama I lysate compared to D420 lysate (7 vs. 3 bacterial peptides). These data illustrate strain-to-strain variability for HLA-DR presentation of some bacterial proteins, while the OmpA-derived peptides were HLA-DR presented in a strain-independent manner. Table 2 summarizes the source antigens from *B. pertussis* that we found presented by HLA-DR using a DDA method.

**Figure 4:**
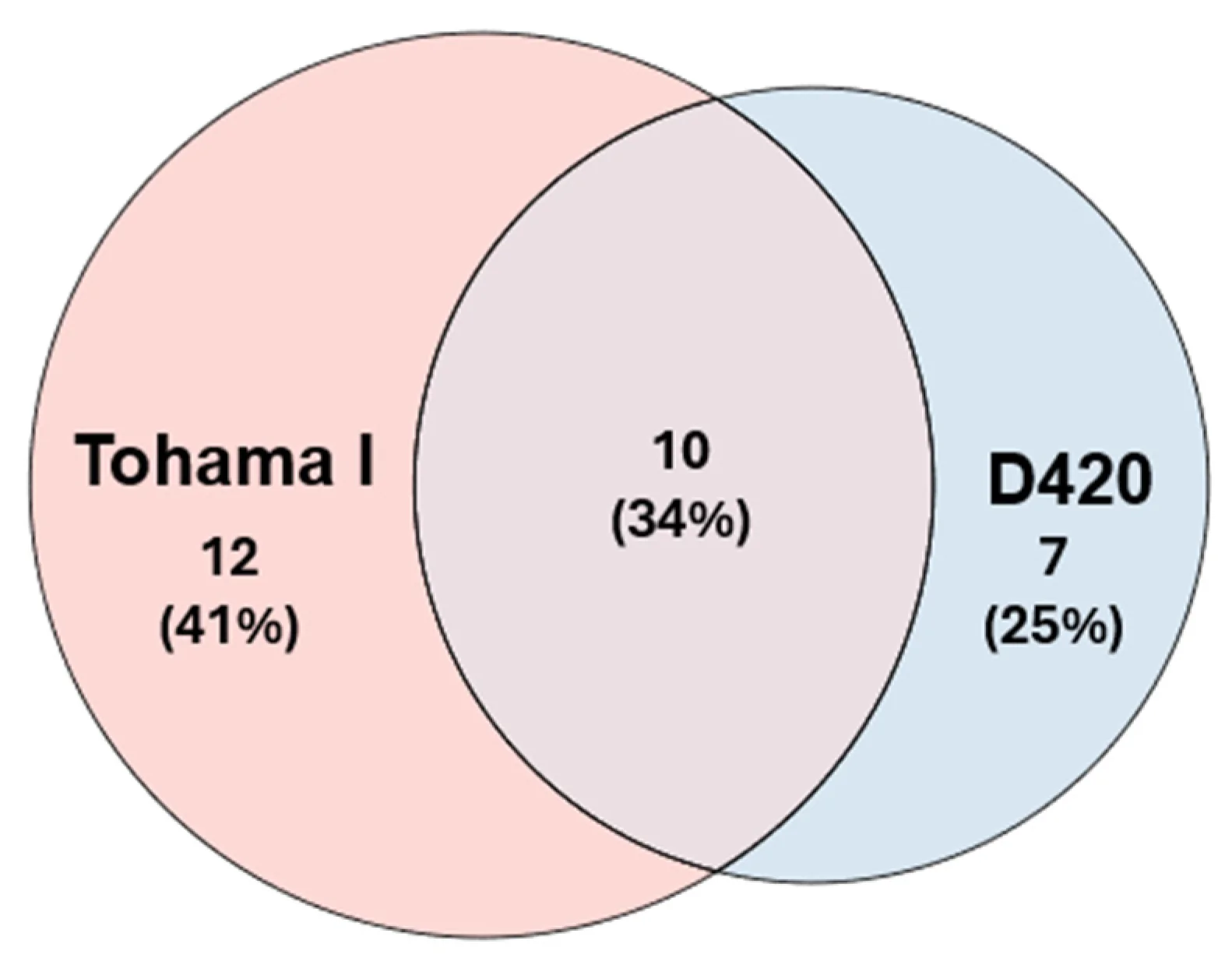
Overlap of *B. pertussis* source proteins that contributed ≥1 HLA-DR ligand after Tohama I versus D420 lysate pulsing of differentiated THP-1 cells.

When data from the Tohama I and D420 strains are combined, 29 source proteins in total were sampled by HLA-DR for peptides with 12 bacterial source proteins unique to Tohama I and 7 bacterial source proteins unique to the D420 strain (Figure 4).

### 3.4. Strain Comparison-Tohama I vs. D420

Unique *B. pertussis* peptide sequences were more numerous for Tohama I than D420 (Figure 3a). Collapsing length variants to shared binding cores preserved this pattern (Figure 3b). The OmpA-derived DRB1*15:01 ligands were detected with either lysate, consistent with strain-independent presentation of this source protein under our assay. By contrast, AlcC- and DNA-binding protein-derived DRB1*01:01 ligands were detected only with Tohama I, and GroEL-derived DRB1*01:01 ligands were more numerous with Tohama I (7 sequences) than D420 (3 sequences), illustrating allele- and strain-dependent differences in the recovered ligand set. Combining both strains, HLA-DR sampled ligands from 29 *B. pertussis* source proteins, including 12 proteins only in Tohama I, 7 only in D420, and 10 detected with both strains (Table 2; Figure 4). The DMT family receptor protein, a peptide source protein unique to strain D420, lacks a homologous coding sequence in the Tohama I genome.

### 3.5. Contribution of Individual HLA-DR Allotypes

**Figure 5:**
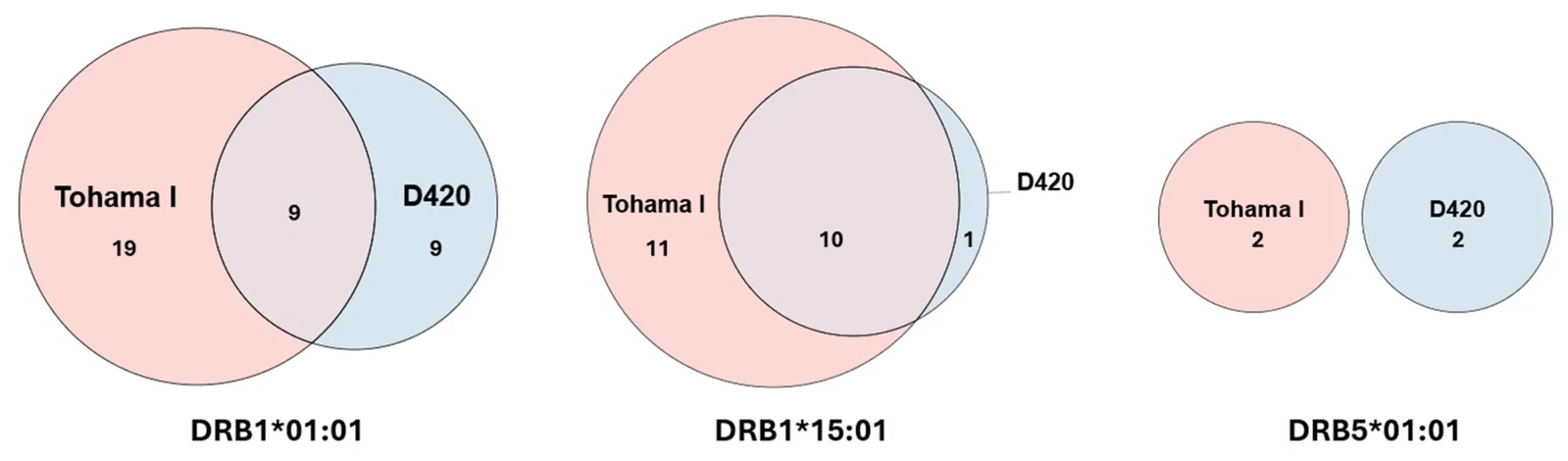
Number of *B. pertussis* peptide ligands assigned to each THP-1 HLA-DR molecule after Tohama I or D420 pulsing.

HLA-DRB1*01:01 contributed 37 bacterial peptide assignments in THP-1 (28 peptides from Tohama I; 18 peptides from D420), as shown in Figure 5.

**Figure 6:**
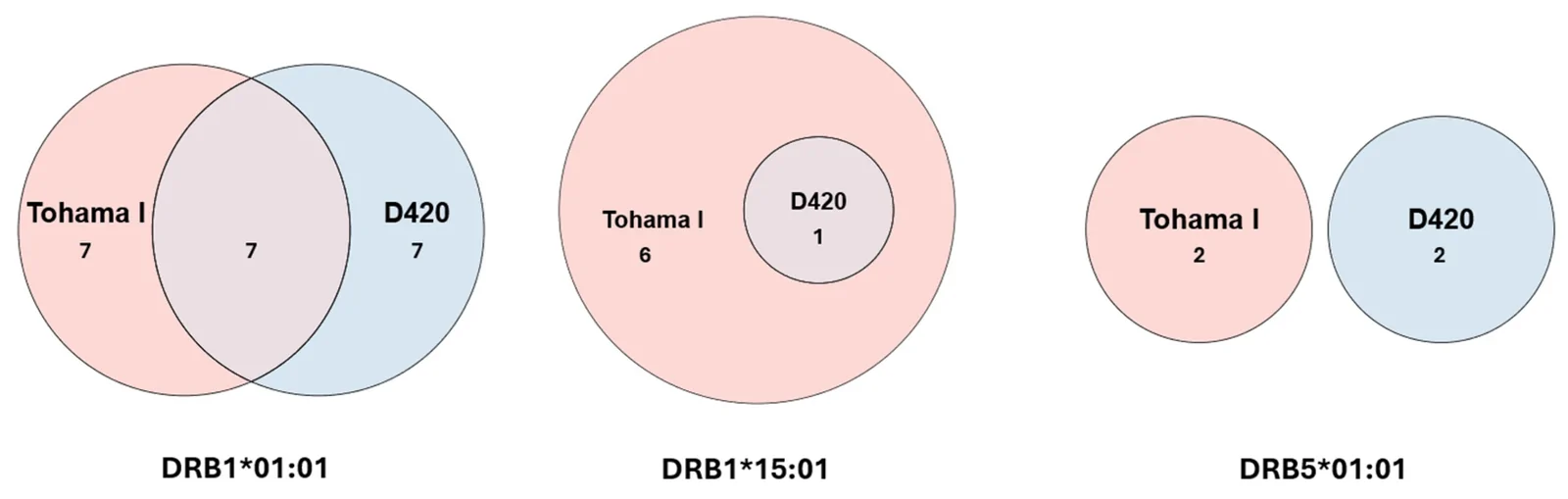
Number of *B. pertussis* source proteins contributing ligands to each THP-1 HLA-DR molecule after Tohama I or D420 lysate pulsing.

At the source-protein level, DRB1*01:01 sampled 14 Tohama I proteins and the same number of D420 proteins, with 7 proteins shared between the two strains (Figure 6).

The DRB1*15:01 allele presented 22 bacterial peptides from 7 Tohama I source proteins, but 11 peptides exclusively from OmpA protein with D420, demonstrating sharp strain-dependent restriction of the recovered ligand repertoire for this allele. HLA-DRB5*01:01 contributed few bacterial ligands overall with no overlap between the two strains (Figure 5 and Figure 6).

### 3.6. Allele Contribution to Bacterial vs. Total DR Immunopeptidome

**Figure 7:**
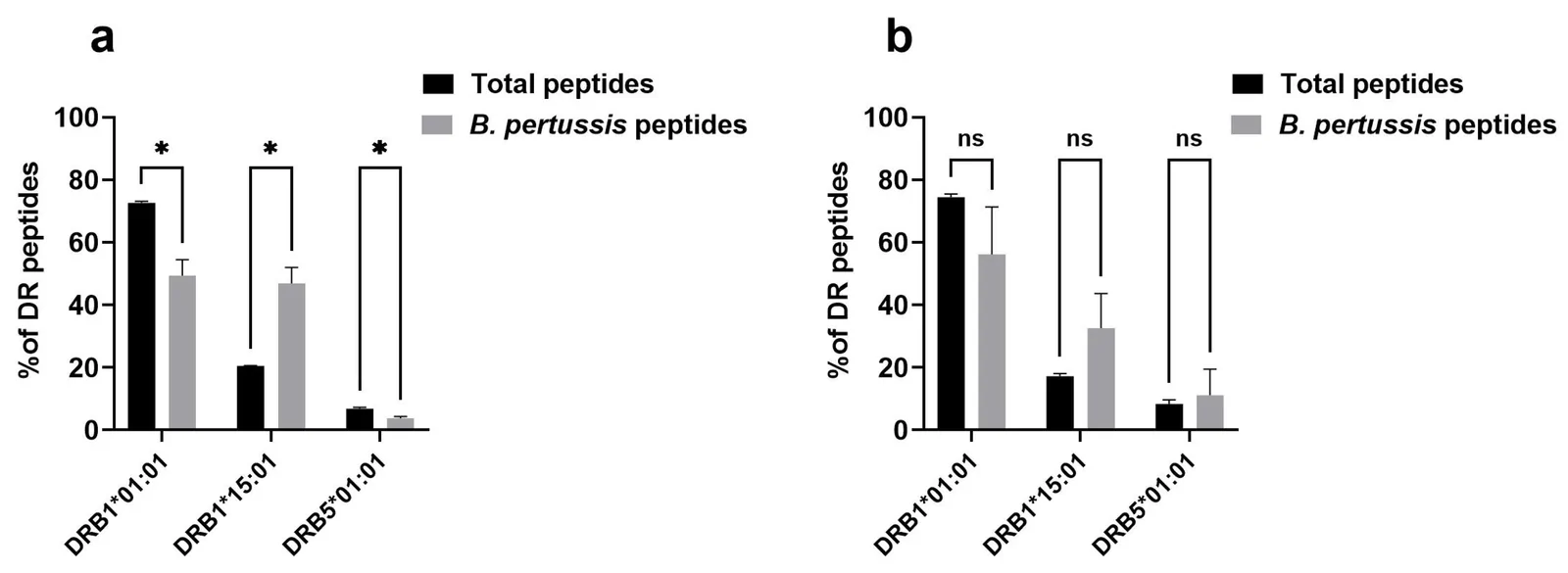
Relative contribution of each THP-1 HLA-DR molecule to (i) the total HLA-DR immunopeptidome and (ii) the *B. pertussis*–derived ligand subset after pulsing with (**a**) Tohama I or (**b**) D420 lysate. Error bars represent the standard deviation (SD) of each group. *: statistically significant (*p* < 0.05), ns: not significant.

Overall, in our experiments with *B. pertussis* strain Tohama I lysates, we found that the contribution of HLA-DRB1*15:01 to the *B. pertussis* peptide set was significantly greater than its contribution to the total DR immunopeptidome (Figure 7a). The same trend was also observed in our experiments with the strain D420 lysates (Figure 7b).

HLA-DRB1*01:01 accounted for the largest share of the total DR immunopeptidome after either lysate pulse (Figure 7). Its contribution to *B. pertussis* ligands was also substantial, consistent with this molecule sampling a broader set of bacterial source proteins rather than a single dominant antigen (Figure 5 and Figure 6). HLA-DRB5*01:01 remained a minor contributor to both the total and bacterial peptide sets. Thus, the allele that dominates the self-ligandome is not necessarily the allele that dominates pathogen-ligand recovery.

## 4. Discussion

We used HLA-DR immunopeptidomics in IFN-γ–treated, PMA-differentiated THP-1 macrophages [33,34,35,36] pulsed with whole *Bordetella pertussis* lysate to define which bacterial proteins yield naturally processed peptide ligands on three HLA-DR allotypes expressed by this line—HLA-DRB1*01:01, HLA-DRB1*15:01, and HLA-DRB5*01:01. Across lysates from the reference strain Tohama I and the clinical isolate D420, we identified 63 HLA-DR–associated bacterial peptide ligands mapping to 29 source proteins. Only 19 ligands from 8 source proteins were detected from both bacterial lysates in this system. Presentation was allele-skewed rather than a single shared inventory: DRB1*01:01 accounted for 37 bacterial ligands from 21 source proteins, DRB1*15:01 for 22 ligands from 7 proteins, and DRB5*01:01 for 4 ligands from 4 proteins. Thirty-two bacterial peptide ligands were uniquely recovered from Tohama I and 12 uniquely from D420.

Together, these results show that *B. pertussis* HLA-DR ligandomes are jointly shaped by bacterial strain and HLA-DR molecule: an antigen that yields peptide ligands for one HLA-DR product often does not for another, and peptides from a given antigen may be recovered after pulse with one strain but not the other. We did not assess T cell recognition; eluted ligands report what reached stable HLA-DR–peptide complexes under the conditions tested, not immunogenicity or protection. A presented peptide may still fail to elicit a CD4^+^ response if the corresponding TCR specificity is missing or deleted (a hole in the repertoire), if precursor frequency is very low, or if regulatory mechanisms dominate. Conversely, CD4 epitopes can exist below the detection limit of data-dependent immunopeptidomics.

A central finding is that DRB1*01:01 and DRB1*15:01 both presented *B. pertussis* ligands, but from different source-protein portfolios. DRB1*01:01 drew ligands from a wide set of bacterial proteins—including GroEL, DNA-binding protein, and AlcC—whereas DRB1*15:01 was dominated by OmpA, particularly when cells were pulsed with D420 lysate, for which all DRB1*15:01assigned bacterial sequences mapped to OmpA. DRB5*01:01 contributed a small, distinct set (four ligands from four proteins, with no bacterial overlap between strains in this allele’s assignments). Most source proteins primarily fed ligands to one of the three HLA-DR molecules assayed, so the alleles did not simply resample the same immunopeptidome at different depths; they recovered ligands from largely non-overlapping antigen sets.

This skew has practical implications for interpreting class II–restricted immunity. HLA-DRB1 expression has been associated with variation in vaccine-elicited antibody responses in population studies [37], and our data add a ligand-level perspective: the DRB1 allele that dominates total DR presentation is not necessarily the allele that dominates presentation of a given pathogen’s ligands. With Tohama I lysate, DRB1*15:01 accounted for a greater fraction of *B. pertussis*–derived DR ligands than of the overall DR immunopeptidome (Figure 7a), and a similar trend was seen with D420 (Figure 7b) but did not reach statistical significance, likely reflecting lower bacterial spectral depth and run-to-run variation for that strain. Together, these patterns support an HLA-DR allele-aware analysis of *B. pertussis* class II presentation rather than collapsing a study across all DR molecules or haplotypes.

Stenger et al. eluted HLA-DR ligands from human monocyte-derived dendritic cells after uptake of *B. pertussis* biomass and linked selected ligands to CD4^+^ T cell responses, reporting a comparatively small set of bacterial sequences [38]. Our workflow differs in APC type (THP-1–derived macrophages), activation (IFN-γ), antigen format (defined lysate pulse), strains (Tohama I and D420), mass spectrometry platform, search and deconvolution pipeline, and the explicit comparison of three DR allotypes in one genotype. Our findings agree qualitatively with prior work that only a subset of bacterial proteins contribute detectable class II ligands in a given model, and that the eluted pool is not dominated exclusively by canonical aP components under the conditions tested.

Separately, genome-wide and donor-based CD4^+^ T cell screens document broad recognition of the *B. pertussis* proteome, including many proteins beyond aP antigens [15,19,39,40]. Those approaches and immunopeptidomics are complementary: recall and screening assays report what memory T cells can recognize across diverse HLA backgrounds, whereas elution reports what is loaded onto defined HLA-DR allotypes in a chosen APC context. In our catalog, the intersection of source proteins with ligand assignments to both DRB1*01:01 and DRB1*15:01 is small—three proteins in total—and includes established aP components pertactin (PRN) and filamentous hemagglutinin (FHA). GroEL-derived ligands were recovered primarily with DRB1*01:01, illustrating that proteins highlighted as immunogenic in population-level T cell studies are not guaranteed to supply ligands on every DR allotype without allele-resolved measurement. None of the eluted sequences in our dataset are currently recorded in the Immune Epitope Database (IEDB); overlap with highly T cell-reactive regions reported for FHA and GroEL in donor cohorts [39] is therefore hypothesis-generating for follow-up tetramer or activation assays in matched HLA types.

Because the bacterial culture, THP-1 differentiation and pulsing, HLA-DR isolation, and mass spectrometry were held constant, comparison of Tohama I and D420 lysates tests how strain choice alters the eluted ligand inventory in a fixed host genotype. Unique bacterial ligand counts were higher for Tohama I than D420 whether analyzed at the level of peptide sequences or collapsed binding cores (Figure 3). OmpA-derived DRB1*15:01 ligands were detected with either lysate, consistent with robust presentation of this source protein in this model. By contrast, AlcC– and DNA-binding protein-derived DRB1*01:01 ligands were seen only with Tohama I, and GroEL-derived DRB1*01:01 ligands were more numerous with Tohama I than D420. Combining both strains, HLA-DR sampled ligands from 29 source proteins: 12 Tohama I–only, 7 D420-only, and 10 detected with both strains (Table 2; Figure 4). That overlap defines a strain-transcendent core while preserving strain-private ligand sets.

An instructive case is PRN. With Tohama I, we detected two distinct PRN-derived ligands—AAGVAAMQGAVVHLQR (presented by DRB1*01:01) and DGWFLEPQAELAVFRAGGGAY (presented by DRB1*15:01)—whereas with D420 lysate neither DRB1 allele yielded a PRN assignment in our experiments, despite D420 encoding a full-length PRN and identical aminoacid sequence at the mapped binding cores relative to Tohama I. PRN-deficient circulating isolates have been reported in aP-using settings [12,41], but that genetic context does not explain the Tohama I–positive, D420-negative pattern here. We favor explanations involving PRN abundance in lysate, competition with the self-ligandome, routing or processing differences, or DDA undersampling—without implying that D420 cannot yield these ligands under other conditions. More generally, absence from an eluate list is not proof of non-presentation; positive calls in one strain/allele combination are nevertheless informative for catalog building. The DMT family transporter, detected only with D420, lacks a homologous coding sequence in the Tohama I reference genome and illustrates how reliance on a historical vaccine strain alone could miss ligand sources relevant to contemporary isolates.

The introduction framed a tension between narrow aP antigen content and an evolving pathogen; the ligand data sharpen that discussion in HLA-specific terms. If most *B. pertussis* source proteins in lysate-pulsed macrophages feed ligands primarily to a single DRB1 allele (with DRB5 contributing only a minor pathogen set), subunit vaccines built from proteins that are poorly represented across common HLA-DR allotypes may have inherently uneven population coverage for CD4^+^ priming, even when those proteins are immunogenic by other readouts. Reformulating aP content remains constrained by manufacturing, safety, and regulatory precedent; our results suggest that the prioritization of bacterial proteins should include allele-resolved antigen presentation data, not binding prediction or T cell screens alone.

Against that background, a carefully circumscribed but notable observation is that PRN and FHA—both established aP components—were among the very few source proteins supplying ligand assignments to both DRB1*01:01 and DRB1*15:01. Ligand elution is not a readout of protective CD4^+^ help, yet under these conditions two priority vaccine antigens also rank among the more cross–DRB1-accessible *B. pertussis* proteins in the eluted ligand data. A parsimonious interpretation is that current aP formulations include representatives of a scarce class of antigens compatible with at least this pair of common DRB1 alleles in macrophages; identifying additional proteins with similar multi-allelic presentation may be non-trivial and may require broader experimental systems than the present study.

Beyond PRN and FHA, our inventory nominates BrkA, OmpA, tracheal colonization factor (TcfA), and the DMT family transporter—together with numerous additional source proteins in Table 1 and Table 2—for follow-up immunopeptidomics across a wider panel of HLA-DR, -DP, and -DQ molecules (L243 captures DR only). Proteomics and immunoproteomics studies have independently highlighted several of these protein classes as immunologically relevant [19,21,27]; the present data add direct evidence of natural HLA-DR ligand formation for selected sequences in a macrophage lysate-pulse model. Whether these ligands support productive CD4^+^ responses in human donors remains to be tested by HLA-matched functional assays.

Several limitations bound interpretation. First, THP-1 macrophages are a tractable human model but represent a single HLA genotype and one differentiation state; dendritic cells, primary macrophages, and other phagocytes may differ in routing and ligand hierarchy. Second, lysate pulse is not infection; it standardizes antigen encounter, but bypasses live bacterial gene expression, adhesin-mediated uptake, and intracellular niches. Third, HLA-DR assignment relied on MHCMotifDecon deconvolution in a heterozygous line; closely related sequences and co-immunoprecipitated DR molecules introduce assignment uncertainty, and HLA-DP and HLA-DQ ligands were not captured. Fourth, DDA with complex lysates favors detection of abundant ligands; strain and allele differences in recovery should be distinguished from differences in presentation capacity where possible (e.g., targeted MS, normalized input, additional replicates). Priority next steps include extending the allele panel and APC types, comparing infection-based antigen delivery with lysate pulse, adding clinical isolates beyond D420, and linking eluted ligands to CD4^+^ recognition in HLA-typed cohorts. Systematic expansion along those axes will clarify which bacterial proteins reliably supply ligands on multiple class II molecules—and whether such patterns correlate with durable helper responses relevant to next-generation pertussis vaccines.

## 5. Conclusions

In this study we completed HLA-DR immunopeptidomics using IFN-γ-stimulated, PMA-differentiated THP-1 macrophages pulsed with whole-cell lysates of *Bordetella pertussis* to define which bacterial proteins yield naturally processed peptide ligands on three HLA-DR allotypes expressed by this line—HLA-DRB1*01:01, HLA-DRB1*15:01, and HLA-DRB5*01:01—and how that inventory differs between the vaccine/reference strain Tohama I and the clinical isolate D420. We recovered 63 bacterial HLA-DR ligands from 29 source proteins. Presentation was allele-skewed: most source proteins contributed ligands primarily to one of the three HLA-DR molecules, so an antigen that supplies peptides to one DR product need not supply peptides to another. Strain further partitioned the ligandome, with only a minority of ligands recovered after pulse with both Tohama I and D420.

Two established acellular pertussis (aP) vaccine antigens, pertactin and filamentous hemagglutinin, were among the few proteins that contributed ligands to both DRB1*01:01 and DRB1*15:01 under these conditions, while other bacterial proteins not in current aP vaccines—including BrkA, OmpA, tracheal colonization factor, and a DMT family transporter—yielded HLA-DR ligands of interest. Eluted ligands report stable cell surface HLA-DR–peptide complexes in this macrophage lysate-pulse model; a priori, they do not establish CD4^+^ immunogenicity or protection. Within that scope, the data support HLA-DR- and strain-aware interpretation of *B. pertussis* class II presentation and argue that prioritization of antigens for next-generation pertussis vaccines should incorporate allele-resolved ligand discovery alongside conventional immunogenicity screens.

## Figures and Tables

**Table 1:** Peptide sequences and the corresponding source proteins from *B. pertussis* presented by HLA-DR of differentiated THP-1 cells.

Sequence	Strain *	Binding Core	HLA	Accession	Source Protein
KGELQPIASNKTREG	T & D	LQPIASNKT	DRB1*15:01	Q7VZG6	Outer membrane protein A
GKGELQPIASNKTREG	T & D				
GKGELQPIASNKTRE	D				
KGELQPIASNKTREGR	T & D				
EGKGELQPIASNKTREG	T & D				
KGELQPIASNKTRE	T & D				
GKGELQPIASNKTREGR	T & D				
EGKGELQPIASNKTRE	T & D				
EGKGELQPIASNKTREGR	T & D				
KGELQPIASNKTREGRA	T				
GKGELQPIASNKTR	T				
KGELQPIASNKTR	T				
TEGKGELQPIASNKTREGR	T				
EGKGELQPIASNKTREGRA	T & D				
EGKGELQPIASNKTR	T & D				
IYTEGKGELQPIASNKTREGR	T				
LKDKFENIGAQLVKD	T	FENIGAQLV	DRB1*01:01	P48210	Chaperonin GroEL
KDKFENIGAQLVKDVA	T & D				
LKDKFENIGAQLVKDVA	T & D				
LKDKFENIGAQLVK	T				
DKFENIGAQLVK	T				
LEDIAILTGGTVIS	T & D	IAILTGGTV	DRB1*01:01		
DIAILTGGTVIS	T				
APRWLAAEEAAGAKRD	T	LAAEEAAGA	DRB1*01:01	Q79GJ5	DNA-binding protein (Histone)
APRWLAAEEAAGAKR	T				
APRWLAAEEAAGAKRDS	T				
GKAPRWLAAEEAAGAKRD	T				
GKAPRWLAAEEAAGAKR	T				
GKAPRWLAAEEAAGAKRDS	T				
DYHGYAPEAATPVR	T	YAPEAATPV	DRB1*01:01	Q7VW17	Alcaligin biosynthesis protein C
YHGYAPEAATPVR	T				
EDYHGYAPEAATPVR	T				
AEDYHGYAPEAATPVR	T				
AGHDIHIINSAKLENTG	T	IHIINSAKL	DRB1*15:01	P12255	Filamentous hemagglutinin
IDSMTALGAIGVQAG	D	MTALGAIGV	DRB1*01:01		
AAGVAAMQGAVVHLQR	T	VAAMQGAVV	DRB1*01:01	P14283	Pertactin autotransporter
DGWFLEPQAELAVFRAGGGAY	T	LAVFRAGGG	DRB1*15:01		
IPVAVALESGALARGDI	T & D	VALESGALA	DRB1*01:01	Q45340	BrkA autotransporter
IPVAVALESGALARG	D				
IDALTVVQGNAARLS	D	LTVVQGNAA	DRB1*01:01	Q7VZS2	Rhodanese domain-containing protein
QLVQQTDYYAPVR	T	LVQQTDYYA	DRB1*15:01		
ISRIALETGIPVANG	T & D	IALETGIPV	DRB1*01:01	Q7VTN4	6,7-dimethyl-8-ribityllumazine synthase
AISRIALETGIPVANG	D	IALETGIPV	DRB1*01:01		
ADLVYVAQTGVVGAPQ	D	YVAQTGVVG	DRB1*01:01	P65622	Membrane protein insertase YidC
VGKFDLLSGRAIWK	D	FDLLSGRAI	DRB1*01:01	Q7VWL3	Outer membrane protein assembly factor BamB
DATAYAIEGGKLVVT	D	YAIEGGKLV	DRB1*01:01	Q7VT71	Malate synthase G
KPGIAYSILGPVAAAN	D	YSILGPVAA	DRB1*01:01	Q7VX91	Aspartokinase
NPNPEILPEAAHAVR	T	ILPEAAHAV	DRB1*01:01	Q7VZ68	NADP-dependent malic enzyme
TPSDQYQFISGTIVR	T & D	YQFISGTIV	DRB1*01:01	Q7VTP4	Phosphopantetheine adenylyltransferase
EPQPEIVPAAPEPVTPP	T & D	IVPAAPEPV	DRB1*01:01	Q7VYR6	SMC-Scp complex subunit ScpB
DGGQVKIIENAEGART	T	VKIIENAEG	DRB1*15:01	Q7VVY2	Chaperone protein DnaK
IPAFSVDEAVAAAEK	T & D	FSVDEAVAA	DRB1*01:01	Q7VVU5	Succinate-CoA ligase [ADP-forming] subunit beta
LPPELAALLAASVEH	T & D	LAALLAASV	DRB1*01:01	Q7VUY8	Integral membrane protein
ASSGLYIAWRATSRR	D	YIAWRATSR	DRB5*01:01	WP_080265467.1	DMT family transporter
AAPPLAHAAAAPVA	T	LAHAAAAPV	DRB1*01:01	Q7VS71	Two-component response regulator
IAGDLCIYTNQNHVIE	T	LCIYTNQNH	DRB1*15:01	Q7VUK0	ATP-dependent protease subunit HslV
QRLLELRARLRRPV	T	LLELRARLR	DRB5*01:01	Q7VU64	Anthranilate synthase component 1
EALNKSQLIAYLVENTGVEAK	T	LIAYLVENT	DRB1*15:01	Q7VWY4	DNA-binding protein Bph2
APLAALAALWLA	D	LAALAALWL	DRB1*01:01	Q7VWT5	Membrane protein
VYLEPGEAAITNTAT	T	LEPGEAAIT	DRB1*01:01	Q7VZY7	Carboxynorspermidine/carboxyspermidine decarboxylase
AGAPAAGVPAAPAPKPAPKP	T	GVPAAPAPK	DRB5*01:01	Q7W0D8	Exported protein
IATLPGIGRSTAAAIA	T	IGRSTAAAI	DRB1*01:01	Q7VST7	Adenine DNA glycosylase
LTPVAGGVWGRAFGRRQDVD	D	WGRAFGRRQ	DRB5*01:01	Q79GX8	Tracheal colonization factor

* T: Tohama I, D: D420.

**Table 2:** Source protein of *B. pertussis*-derived ligands presented by HLA-DR of differentiated THP-1 cells.

Accession	Protein Name	Number of Peptides	Strain *
Q7VZG6	Outer membrane protein A	16	T & D
P48210	Chaperonin GroEL	7	T & D
Q79GJ5	DNA-binding protein (Histone)	6	T
Q7VW17	Alcaligin biosynthesis protein C	4	T
P12255	Filamentous hemagglutinin ^†^	2	T & D
P14283	Pertactin autotransporter ^†^	2	T
Q45340	BrkA autotransporter	2	T & D
Q7VZS2	Rhodanese domain-containing protein	2	T & D
Q7VTN4	6,7-dimethyl-8-ribityllumazine synthase	2	T & D
P65622	Membrane protein insertase YidC	1	D
Q7VWL3	Outer membrane protein assembly factor BamB	1	D
Q7VT71	Malate synthase G	1	D
Q7VX91	Aspartokinase	1	D
Q7VZ68	NADP-dependent malic enzyme	1	T
Q7VTP4	Phosphopantetheine adenylyltransferase	1	T & D
Q7VYR6	SMC-Scp complex subunit ScpB	1	T & D
Q7VVY2	Chaperone protein DnaK	1	T
Q7VVU5	Succinate-CoA ligase	1	T & D
Q7VUY8	Integral membrane protein	1	T & D
WP_080265467.1	DMT family transporter	1	D
Q7VS71	Two-component response regulator	1	T
Q7VUK0	ATP-dependent protease subunit HslV	1	T
Q7VU64	Anthranilate synthase component 1	1	T
Q7VWY4	DNA-binding protein Bph2	1	T
Q7VWT5	Membrane protein	1	D
Q7VZY7	Carboxynorspermidine/carboxyspermidine decarboxylase	1	T
Q7W0D8	Exported protein	1	T
Q7VST7	Adenine DNA glycosylase	1	T
Q79GX8	Tracheal colonization factor	1	D

* T: Tohama I, D: D420, ^†^ Included in acellular pertussis vaccines.

## Data Availability

The raw mass spectrometry data acquired in this study and result files are openly available in the Center for Computational Mass Spectrometry (MassIVE, https://massive.ucsd.edu, accessed on 27 July 2026) under MSV000102625 accession number.

## Abbreviations

The following abbreviations are used in this manuscript:

ACN	Acetonitrile
AlcC	Alcaligin biosynthesis protein
aP	Acellular pertussis vaccine
APC	Antigen-presenting cell
BG	Bordet–Gengou medium
*B. pertussis*	*Bordetella pertussis*
BrkA	Bordetella resistance to killing A
CD	Cluster of differentiation
CID	Collision-induced dissociation
DMT	Divalent metal transporter
FBS	Fetal bovine serum
DDA	Data-dependent acquisition
FDR	False discovery rate
FHA	Filamentous hemagglutinin
FIM	Fimbrial antigen
FITC	Fluorescein isothiocyanate
HLA	Human leukocyte antigen
IDA	Information-dependent analysis
IEDB	Immune epitope database
IFN-γ	Interferon gamma
iRT	Indexed retention time
MS	Mass spectrometry
OmpA	Outer membrane protein A
PBS	Phosphate-buffered saline
PE	Phycoerythrin
PMA	Phorbol 12-Myristate Acetate
PRN	Pertactin
PT	Pertussis toxin
PTM	Post-translational modification
RP-HPLC	Reverse-phase high-performance liquid chromatography
RPMI	Roswell Park Memorial Institute
SS	Stainer-Scholte medium
TcfA	Tracheal colonization factor
TFA	Trifluoroacetic acid
Th	T helper
TOF	Time of flight
UV	Ultraviolet
wP	Whole-cell pertussis vaccine
